# Sugar transporters: mediators of carbon flow between plants and microbes

**DOI:** 10.3389/fpls.2025.1536969

**Published:** 2025-04-16

**Authors:** Mengyu Lei, Xiaodi Wang, Kuan Chen, Qianqian Wei, Miaomiao Zhou, Gong Chen, Shuai Su, Yuying Tai, Kexin Zhuang, Dexiao Li, Mengjuan Liu, Senlei Zhang, Youning Wang

**Affiliations:** ^1^ State Key Laboratory for Crop Stress Resistance and High-Efficiency Production, College of Agronomy, Northwest A&F University, Yangling, China; ^2^ State Key Laboratory of Crop Genetic Improvement, College of Plant Science and Technology, Huazhong Agricultural University, Wuhan, China

**Keywords:** sugar transporters, plant-microbial interaction, function, molecular mechanism, pathogen invasion, symbiosis

## Abstract

Pathogens and symbiotic microorganisms significantly influence plant growth and crop productivity. Enhancing crop disease resistance and maximizing the beneficial role of symbiotic microorganisms in agriculture constitute critical areas of scientific investigation. A fundamental aspect of plant-microorganisms interactions revolves around nutritional dynamics, characterized by either “food shortage” or “food supply” scenarios. Notably, pathogenic and symbiotic microorganisms predominantly utilize photosynthetic sugars as their primary carbon source during host colonization. This phenomenon has generated substantial interest in the regulatory mechanisms governing sugar transport and redistribution at the plant-microorganism interface. Sugar transporters, which primarily mediate the allocation of sugars to various sink organs, have emerged as crucial players in plant-pathogen interactions and the establishment of beneficial symbiotic associations. This review systematically categorized plant sugar transporters and highlighted their functional significance in mediating plant interactions with pathogenic and beneficial microorganisms. Furthermore, we synthesized recent advancements in understanding the molecular regulatory mechanisms of these transporters and identified key scientific questions warranting further investigation. Elucidating the roles of sugar transporters offers novel strategies for enhancing crop health and productivity, thereby contributing to agricultural sustainability and global food security.

## 1 Introduction

In natural ecosystems, plants engage in intricate interactions with various microorganisms inhabiting their phyllosphere and rhizosphere, ranging from antagonistic encounters with pathogens to mutualistic symbioses with beneficial microbes. While plant defense mechanisms against pathogens have been extensively studied (Jones et al., 2024; Ngou et al., 2022), beneficial microorganisms, such as plant growth-promoting rhizobacteria (PGPR), *arbuscular mycorrhizal* (AM) fungi and rhizobia that enhance nutrient acquisition, stress resilience, and growth in their hosts (Glick and Gamalero, 2021; Udvardi and Poodle, 2013; Wipf et al., 2019), deserve greater attention. For instance, *Bacillus subtilis* (BS) promotes plant growth and stress tolerance (Arnaouteli et al., 2021; Gouda et al., 2018), the host plant exchanges nutrients with AM fungi for mutual benefit (Wipf et al., 2019), while rhizobia-legume symbioses enable biological nitrogen fixation (Udvardi and Poodle, 2013).

Carbohydrates, as primary energy sources, play a central role in these interactions. Likewise, pathogens depend on host-derived sugars for survival, sparking competition for carbon resources during infection (Bezrutczyk et al., 2018; Naseem et al., 2017). In contrast, symbiotic relationships involve cooperative carbon allocation, where plants supply sugars to mutualistic microbes in exchange for nutrients or protection (Bezrutczyk et al., 2018; Tian et al., 2021; Udvardi and Poodle, 2013; Wipf et al., 2019). Sugar availability critically influences plant-pathogen dynamics, directly impacting host resistance (Bolouri Moghaddam and Van den Ende, 2012). Similarly, sugar is indispensable during the early stages of symbiotic interactions (Loo et al., 2024; Tian et al., 2021), and is vital for nitrogen-fixing bacteroids in mature root nodules (Liu et al., 2018; Udvardi and Poodle, 2013). Despite emerging insights, the mechanisms governing carbon exchange in plant-microbe interactions remain poorly understood, underscoring the need to elucidate nutrient-provisioning strategies, particularly sugar-related pathways.

The translocation of sugars from source to sink tissues is mediated by specialized transporters, including Monosaccharide Transporters (MSTs), H^+^/sucrose transporters (SUTs), and Sugars Will Eventually be Exported Transporters (SWEETs) (Braun, 2022; Chen et al., 2015a, 2024). These transporters not only regulate carbon partitioning within plants but also modulate interactions with microorganisms (Breia et al., 2021; Chen et al., 2024; Geiger, 2020). Following the classification of sugar transporters, this review highlights recent advances in their molecular mechanisms during plant immunity and plant-beneficial microorganism interactions, providing a foundation for future research aimed at harnessing these pathways for sustainable agriculture.

## 2 Sugar transporters

### 2.1 Monosaccharide transporters

**Figure 1:**
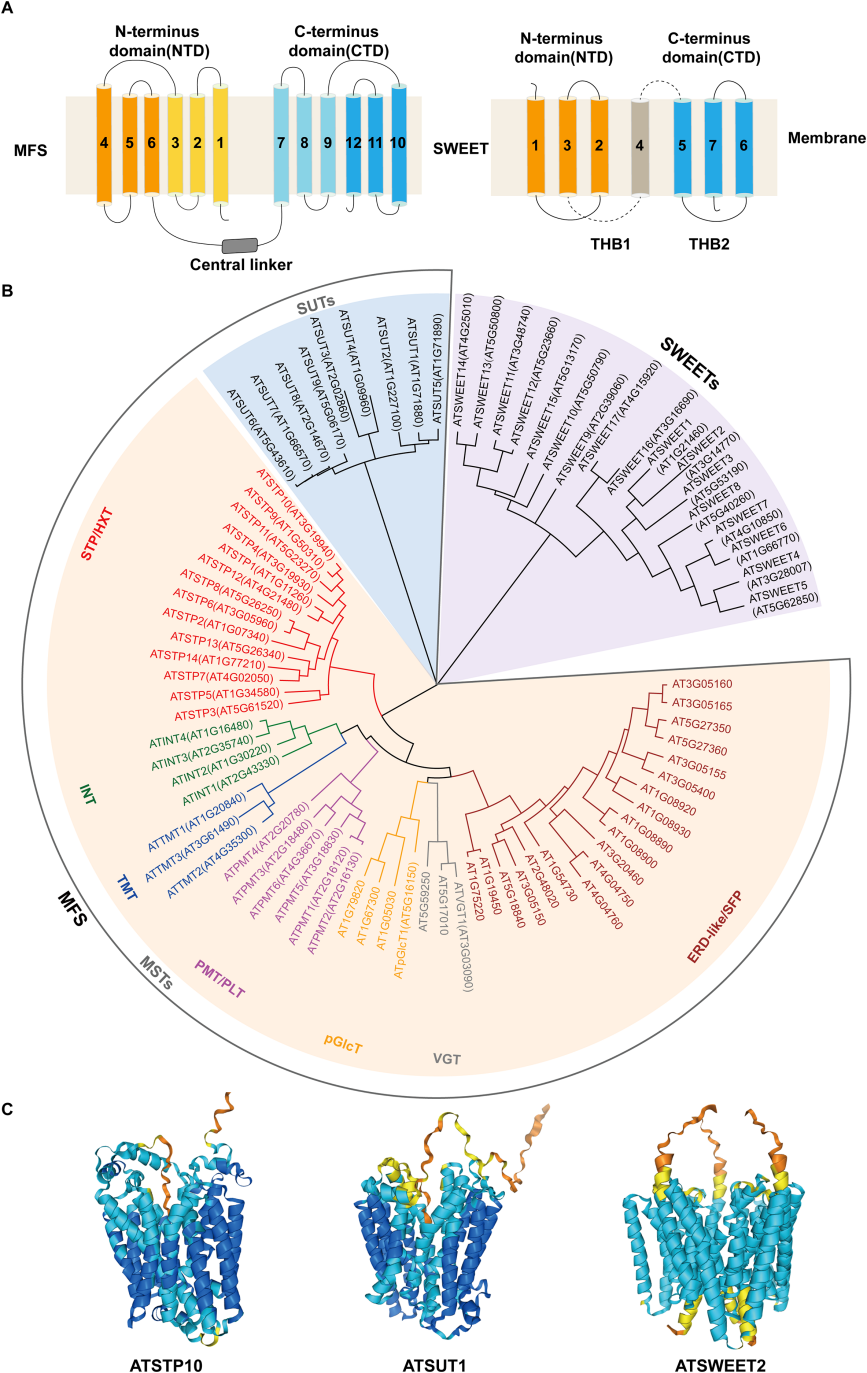
The structures and classifications of MFS and SWEET. **(A)** Topological structures of MFS and SWEET. The MFS topology consists of 12 transmembrane (TM) segments forming two six-helix bundles, each with inverted 3-TM repeats. The N-terminus (TM1-6; light orange/orange) and C-terminus (TM7-12; light blue/blue) are connected by cytoplasmic rings (gray). The SWEET topology includes 7 transmembrane helices (TMH) arranged in a 3-1-3 structure: two 3-TMH MtN3 motifs (TMH1-3, orange; TMH5-7, blue) connected by TMH4. Each MtN3 motif forms triple helix bundles (THBs) with the arrangement TMH1-TMH3-TMH2/TMH5-TMH7-TMH6. **(B)** A phylogenetic tree of MFS and SWEET in Arabidopsis. The phylogenetic tree shows SWEET family members (light purple background) and MFS family members. MFS members are divided into SUT (light blue background) and MST (yellow background). MST subfamilies are labeled in red (STP/HXT), green (INT), blue (TMT), magenta (PMT/PLT), orange (pGlcT), gray (VGT), and brown (ELD-like/SFP). **(C)** Structural model predictions of MFS (AtSTP10 and AtSUT1) and SWEET (AtSWEET2) in Arabidopsis. AtSTP10 and AtSUT1 have standard MFS structures, while AtSWEET2 has a typical SWEET homotrimeric structure.

MSTs in plants are H^+^-coupled symporters localized to the cell membrane, belonging to the Major facilitator superfamily (MFS) and containing 12 transmembrane domains (**Figure 1A**) (Niño-González et al., 2019; Paulsen et al., 2019). These transporters utilize the proton gradient generated by plasma membrane H^+^-ATPase to actively transport monosaccharides against concentration gradients (Niño-González et al., 2019; Paulsen et al., 2019).

In Arabidopsis (*Arabidopsis thaliana*), 53 MST members have been identified (**Figure 1B**), phylogenetically classified into seven clades: Sugar Transport Protein (STP), Polyol/Monosaccharide Transporter (PMT/PLT), Tonoplast Membrane Transporter (TMT), Inositol Transporter (INT), Vacuolar Glucose Transporter (VGT), Plastidic Glucose Transporter (pGlcT), and Early Response to Dehydration Six-Like (ERD-like/SFP) (Büttner, 2007; Johnson and Thomas, 2007). Among these, the ERD-like/SFP and STP subfamilies are the largest, comprising 19 and 14 members, respectively (**Figure 1B**).

Most MSTs exhibit broad substrate specificity, transporting multiple monosaccharides with varying affinities (Büttner, 2010; Geiger, 2020). For instance, AtSTP1 in Arabidopsis shows a high affinity for glucose, while AtSTP6 and AtSTP13 preferentially transport fructose, albeit with residual activity toward galactose, mannose, xylose, and other pentoses (Büttner, 2010). A subset of MSTs, however, display substrate specificity: AtSTP9 is glucose-specific, and AtSTP14 is galactose-specific (Poschet et al., 2010; Schneidereit et al., 2003). Functionally, following phloem unloading and enzymatic hydrolysis of sucrose into glucose and fructose, MSTs mediate monosaccharide uptake into sink tissues (Geiger, 2020). They are pivotal in monosaccharide absorption, distribution, utilization, and storage, thereby orchestrating plant growth and development.

### 2.2 Sucrose transporters

SUTs, also known as sucrose/H^+^ symporters (SUCs), are key players in sucrose translocation across plant membranes (**Figure 1C**) (Sauer, 2007). SUTs belong to the MFS but are phylogenetically distinct from MSTs (**Figure 1B**). They utilize ATP-dependent proton gradients to drive sucrose transport, particularly during phloem loading (Sauer, 2007).

Phylogenetically, SUTs are classified into three types: Type I, Type II, and Type III (Reinders et al., 2012). Type I SUTs, which are exclusive to eudicots and localized to the plasma membrane, exhibit high substrate affinity and are critical for phloem loading, ensuring efficient distribution of photoassimilates (Cai et al., 2021; Lasin et al., 2020; Tong et al., 2022). Type II SUTs, characterized by low substrate affinity, are further subdivided into dicot-specific Type IIA and monocot-specific Type IIB (Reinders et al., 2012). Type IIA SUTs, found in early vascular plants (e.g., *Selaginella*) and mosses, participate in phloem loading but may have overlapping roles with other SUTs in fine-tuning sucrose transport (Reinders et al., 2012). In monocots, Type IIB SUTs replace Type I SUTs for phloem loading (Baker et al., 2016; Wang et al., 2021a) and additionally regulate phloem unloading and sucrose pool organization (Sun et al., 2022). Type III SUTs, which are ubiquitous in terrestrial plants, exhibit intermediate substrate affinity (Reinders et al., 2012). They are located on the vacuolar membrane or plasma membrane, or both, and can regulate the storage and distribution of sucrose in cells, thereby participating in plant growth and development (Eom et al., 2011; Garg et al., 2022; Leach et al., 2017; Liang et al., 2023; Wang et al., 2016). Collectively, SUTs orchestrate sucrose dynamics from source to sink tissues, underpinning carbon allocation and plant productivity.

### 2.3 Sugars will eventually be exported transporters

SWEETs represent a unique class of sugar transporters distinct from MSTs and SUTs and belong to the *Medicago truncatula* Nodulin3-like (MtN3-like) clan, which is different from the MFS superfamily (**Figure 1B**) (Xuan et al., 2013). SWEETs are characterized by seven α-helical transmembrane domains and mediate the passive diffusion of sugars along concentration gradients without requiring energy consumption (**Figure 1A**) (Han et al., 2017; Tao et al., 2015). Structurally, plant SWEETs typically form homotrimeric complexes, a conformation that is critical for their transport activity (**Figure 1C**) (Han et al., 2017; Tao et al., 2015). Functionally, SWEETs are indispensable for phloem sugar unloading, sucrose efflux, and bidirectional sugar transport across membranes (Braun, 2022; Breia et al., 2021).

Plant SWEETs are phylogenetically divided into four clades (Clades I-IV), though sequence homology is relatively low (Eom et al., 2015). While clade membership partially correlates with substrate preference, functional predictions remain challenging. Generally, Clade I and Clade II SWEETs transport hexose, Clade III SWEETs primarily mediate the transport of sucrose, and Clade IV SWEETs are associated with the transport of fructose (Chen et al., 2024; Eom et al., 2015). Subcellular localization further distinguishes these clades: Clade IV SWEETs localize to the tonoplast, while most others reside in the plasma membrane (Chen et al., 2024; Eom et al., 2015). The diversity in substrate specificity and subcellular localization enables SWEETs to regulate multiple physiological processes (Chen et al., 2015b; Kryvoruchko et al., 2016; Le Hir et al., 2015; Valifard et al., 2021).

## 3 Sugar transporters as key players in plant-pathogen dynamics

In plant-pathogen dynamics, where plants are engaged in an ongoing battle with pathogenic microorganisms, sugar transporters play a pivotal role (**Table 1**) (Chen et al., 2024; Devanna et al., 2021). These transporters, which are vital for plant metabolism, can help plants resist pathogens (Chen et al., 2015b; Kim et al., 2021; Li et al., 2017; Sade et al., 2013; Yamada et al., 2016; Yamada and Mine, 2024). However, pathogens often manipulate these transporters to obtain carbon sources for their survival (Chen et al., 2010; Cohn et al., 2014; Cox et al., 2017; Elliott et al., 2024; Huai et al., 2019, 2020, 2022; Liu et al., 2024; Milne et al., 2019; Sun et al., 2021). A deeper comprehension will lay a foundation for enhancing plants’ natural defense mechanisms against pathogenic invasions at the molecular level.

**Table 1:** Role of various sugar transporters identified in plants.

Name	Organism	Gene Function	References
*AtSTP8*	*Arabidopsis thaliana*	• Exhibits a wide range of hexose transport activity • Overexpression of *AtSTP8* promotes resistance to powdery mildew	Liu et al., 2021
*AtSTP13*	*Arabidopsis thaliana*	• Induced by the flg22 peptide of bacterial flagellin • Promotes the uptake of hexose in apoplast and isolates sugar and pathogens	Yamada et al., 2016
*TaSTP3*	*Triticum aestivum* L.	• Transports both sucrose and hexose • Upregulation of *TaSTP3* increases susceptibility during wheat stripe rust infection • Transcription factors TaWRKY19, TaWRKY61, and TaWRKY82 coordinate its expression	Huai et al., 2022
*TaSTP13*	*Triticum aestivum L.*	• Knockdown of *TaSTP13* enhances wheat resistance to *Puccinia striiformis* f.sp. *tritici* • Overexpression of *TaSTP13* promotes susceptibility to powdery mildew	Huai et al., 2020
*TaSTP6*	*Triticum aestivum L.*	• ABA significantly enhances *TaSTP6* expression • Upregulation of *TaSTP6* contributes to host fungal sugar acquisition and promotes fungal infection	Huai et al., 2019
*LeHT1*	*Solanum lycopersicum* L.	• Expressed in *tomato yellow leaf curl virus* resistant varieties • *leht1* plants show inhibition of growth and enhance virus accumulation and spread	Eybishtz et al., 2010
*ZmSUT1*	*Zea mays* L.	• Expressed in various sink tissues • Loads sucrose in phloem companion cells and retrieves sucrose in other cell types from the apoplasm	Baker et al., 2016	
*OsSWEET11/13*	*Oryza sativa* L.	• Significantly induced by *Rhizoctonia solani* • *Ossweet11/13* plants increase pathogen resistance • Overexpression of *OsSWEET11/13* plants are more susceptible	Gao et al., 2018	
*OsSWEET14*	*Oryza sativa* L.	• Significantly induced by *Rhizoctonia solani* • Overexpression of *OsSWEET14* enhances rice resistance to *Rhizoctonia solani*	Kim et al., 2021	
*MeSWEET10a*	*Manihot esculenta* Crantz	• TAL20Xam668 specifically induces the sugar transporter MeSWEET10a to promote virulence	Cohn et al., 2014	
*CsSWEET1*	*Citrus sinensis* L.	• Induced by the genus *Xanthomonas* • It is susceptible to citrus bacterial canker	Hu et al., 2014	
*GhSWEET10d*	*Gossypium hirsutum* L.	• Activated by *Xcm’s* effector Avrb6 to promote pathogen infection	Cox et al., 2017	
*GhSWEET42*	*Gossypium hirsutum L.*	• Knockdown of *GhSWEET42* decreases glucose content and enhances plants’ resistance to *Verticillium dahliae*	Sun et al., 2021
*AtSWEET2*	*Arabidopsis thaliana*	• *Pythium* infection induces the upregulation of *AtSWEET2* in roots • The *Atsweet2* mutants are more susceptible to the oomycete	Chen et al., 2015b
*SISWEET15*	*Solanum lycopersicum* L.	• *Botrytis cinerea* infection enhances its expression	Asai et al., 2016
*IbSWEET10*	*Ipomoea batatas* L.	• Significantly upregulated by *Fusarium oxysporum* • The *IbSWEET10*-overexpressing plants are more resistant • The *IbSWEET10*-RNAi lines exhibit more susceptibility	Li et al., 2017
*MtSWEET1b*	*Medicago truncatula*	• Strongly upregulated in arbuscule-containing cells • Overexpression of *MtSWEET1b* promotes the growth of intraradical mycelium •Arbuscule-specific overexpression of *MtSWEET1b* ^Y57A/G58D^ can result in AM disintegration	An et al., 2019
*MtSWEET11*	*Medicago truncatula*	• Rhizobia infection significantly induces the expression of *MtSWEET11* in infected root hair cells, meristem, invasion zone, and vasculature of nodules	Kryvoruchko et al., 2016
*LjSWEET3*	*Lotus japonicus* L.	• Gradually upregulated during the development of nodules • Specifically overexpressed in the vascular tissues of mature nodules	Sugiyama et al., 2017
*GmSWEET6*	*Glycine max* L.	• Involved in AM symbiosis • Mediates the efflux of sucrose to fungi	Zheng et al., 2024
*GmSWEET15*	*Glycine max* L.	• The expression is significantly increased in cells colonized by AM fungi	Zhao et al., 2019
*StSWEET1b*	*Solanum tuberosum* L.	• Strongly induced during symbiosis with AM fungi	Manck-Götzenberger and Requena, 2016
*StSWEET7a*	*Solanum tuberosum* L.	• Strongly induced during symbiosis with AM fungi • Overexpression of *StSWEET7a* increases the content of glucose, fructose, and mannose, and promotes colonization of AM fungus *Rhizophagus irregularis*	Tamayo et al., 2022

### 3.1 Sugar transporters: guardians of plant immunity against pathogens

Sugar transporters serve as critical gatekeepers in plant-pathogen interactions, safeguarding host sugar reserves and disrupting pathogen nutrient acquisition (Chen et al., 2024; Devanna et al., 2021). By modulating sugar availability, these transporters enhance plant resistance through two strategies: sequestering sugars to starve pathogens and activating defense signaling pathways (Chen et al., 2015b; Yamada et al., 2016; Yamada and Mine, 2024).

**Figure 2:**
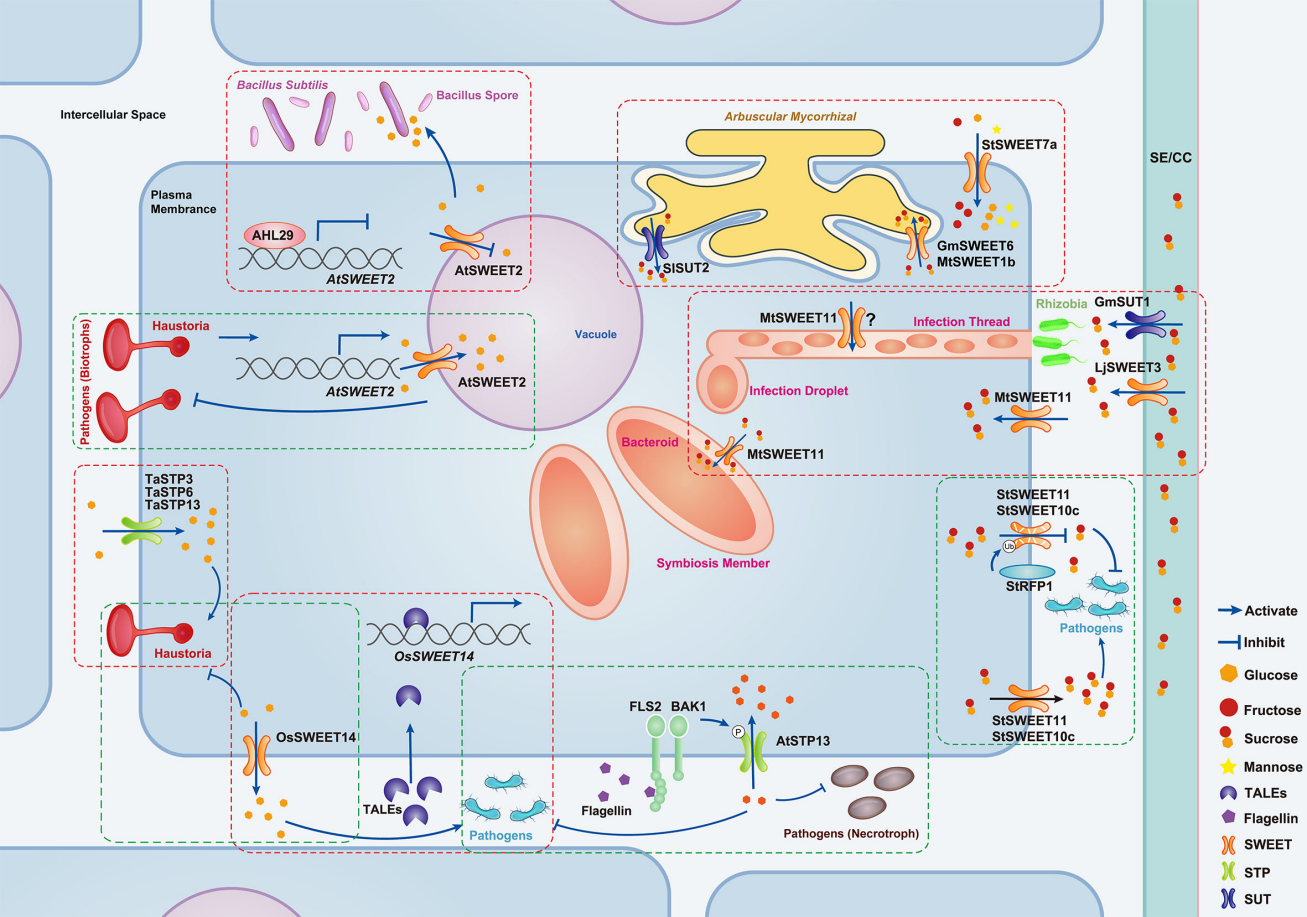
Schematic of sugar transporter functions in plant-microbe interactions. This figure elucidates the pivotal roles of sugar transporters in modulating microbial interactions. In biotrophic fungal infections (e.g., *Puccinia triticina*), TaSTP3, TaSTP6, and TaSTP13 aid pathogen colonization by sugar import (Huai et al., 2019, 2020, 2022; Milne et al., 2019), while AtSWEET2, and OsSWEET14 inhibit it via sugar export to vacuoles or apoplast (Chen et al., 2010, 2015b; Kim et al., 2021). During bacterial or necrotrophic fungal infections (e.g., *Botrytis cinerea*), AtSTP13 limits pathogen growth by depleting apoplastic sugar (Yamada et al., 2016), but OsSWEET14, StSWEET11, and StSWEET10c are exploited to supply apoplastical sugar, boosting pathogen colonization (Chen et al., 2010; Wu et al., 2022, 2024b; Xu et al., 2024). In beneficial interactions, the downregulation of *AtSWEET2* promotes the colonization of beneficial microbes (e.g., *Bacillus subtilis*) (Wu et al., 2024a), and GmSWEET6, MtSWEET1b, StSWEET7a, and SISUT2 manage plant-*arbuscular mycorrhizal* fungus symbiosis (An et al., 2019; Bitterlich et al., 2014; Tamayo et al., 2022; Zheng et al., 2024). Additionally, GmSUT1, MtSWEET11, and LjSWEET3 support legume-rhizobia symbiosis (Deng et al., 2022; Kryvoruchko et al., 2016; Sugiyama et al., 2017). While it has been proposed that MtSWEET11 in infection threads aids sucrose transport, this hypothesis lacks definitive evidence, hence the use of a question mark in the figure (Kryvoruchko et al., 2016). The red dashed box highlights sugar transporters that promote plant-microbe interactions, whereas the green box indicates those that inhibit such interactions.

STPs play a pivotal role in plant defense against pathogens by enhancing cellular sugar uptake. STP-mediated glucose influx increases the intracellular levels of glucose-6-phosphate (G6P), which on one hand inhibits the activity of protein phosphatases (such as ABI1), thereby enhancing the activity of calcium-dependent protein kinase 5 (CPK5) and promoting plant defense responses (Yamada and Mine, 2024). On the other hand, G6P also promotes the biosynthesis of salicylic acid (SA) through a CPK5-independent signaling pathway, thereby coordinating plant immune signaling (Yamada and Mine, 2024). Furthermore, in Arabidopsis, the upregulation of *AtSTP13* during pathogen infection enhances competition for extracellular hexoses, starving pathogens like *Pseudomonas syringae* pv. *tomato* (Pst) DC3000 and *Botrytis cinerea* (gray mold), and limiting their proliferation (**Table 1**, **Figure 2**) (Yamada et al., 2016). In tomato (*Lycopersicon esculentum*), the hexose transporter LeHT1 maintains intracellular glucose homeostasis and hexose/sucrose ratios, which are essential for resisting *Tomato yellow leaf curl virus* (**Table 1**) (Sade et al., 2013).

SWEETs, typically associated with sugar efflux, paradoxically enhance resistance in specific contexts. In rice (*Oryza sativa*), *OsSWEET14* upregulation during *Rhizoctonia solani* (sheath blight) infection reduces apoplastic sugar content, inhibiting pathogen growth (Kim et al., 2021) (**Table 1**; **Figure 2**). Cassava (*Ipomoea batatas*) infected with *Fusarium oxysporum* exhibits elevated *IbSWEET10* expression, correlating with improved fungal resistance (Li et al., 2017). Surprisingly, AtSWEET2 in Arabidopsis confers resistance by sequestering glucose in vacuoles, preventing its efflux to pathogens (**Table 1**, **Figure 2**) (Chen et al., 2015b). These findings highlight that sugar transporters emerge as versatile targets for engineering disease-resistant crops.

### 3.2 Pathogens exploit sugar transporters to facilitate infection

However, not all sugar transporters function solely to resist pathogen invasion. Among them, even as guardians, they can be manipulated by pathogens to varying degrees, facilitating pathogen infection of hosts (Bezrutczyk et al., 2018; Chen et al., 2024). In nature, some biotrophic pathogens can manipulate sugar transport in host cells through MSTs, acquiring sugar via the plant-haustorium interface (Chen et al., 2010, 2024; Huai et al., 2022). For example, *TaSTP13* in wheat (*Triticum aestivum*), a homolog of *AtSTP13*, which enhances plant disease resistance, can elevate the glucose content in leaves, subsequently enhancing the susceptibility of plants to powdery mildew (Huai et al., 2020; Milne et al., 2019). In addition, TaSTP3 and TaSTP6 also increase sugar content in leaves, leading to increased susceptibility of wheat to pathogens (**Table 1**, **Figure 2**) (Huai et al., 2019, 2022).

Moreover, pathogens actively manipulate SWEETs at the transcriptional level to ensure their survival during infection by securing a vital carbon source (**Figure 2**). Infection with *Xanthomonas* spp. in plants such as rice, cassava, and cotton (*Gossypium hirsutum*) can upregulate *SWEET* family genes, facilitating pathogen colonization (Chen et al., 2010; Cohn et al., 2014; Cox et al., 2017). For instance, *Xanthomonas oryzae* pv. *Oryzae* (*Xoo*), the causative agent of bacterial leaf blight in rice, specifically upregulates *OsSWEET11* expression, allowing it to colonize thin-walled cells surrounding leaf vascular bundles (**Table 1**, **Figure 2**) (Chen et al., 2010; Wu et al., 2022; Xu et al., 2024). Similarly, an African strain of *Xoo* stimulates the expression of *OsSWEET14*, driving it to transport glucose to the extracellular space in HEK293T cells and oocytes, which reduces rice resistance against bacterial blight (**Table 1**, **Figure 2**) (Blanvillain-Baufumé et al., 2017; Chen et al., 2010; Streubel et al., 2013). In cassava (*Manihot esculenta*), *Xanthomonas axonopodis* pv. *manihotis* (*Xam*) enhances its virulence by elevating *MeSWEET10a* expression (Cohn et al., 2014). Additionally, blocking the induction of *MeSWEET10a* to reduce cassava susceptibility has been proven feasible (Elliott et al., 2024). In cotton, *Xanthomonas citri* subsp. *malvacearum* (*Xcm*) specifically upregulates *GhSWEET10d*, a sucrose transporter gene, via its effector Avrb6, thereby facilitating pathogen infection of plants (Cox et al., 2017). Furthermore, GhSWEET42 renders plants susceptible to *Verticillium dahliae*, a soil-borne fungal pathogen, through glucose translocation (**Table 1**) (Sun et al., 2021).

Interestingly, STPs and SWEETs can synergistically promote pathogen infection, as shown in the latest research (Liu et al., 2024). The infection of *Erysiphe heraclei* activates HmSWEET8 in *Heracleum moellendorffii* Hance, leading to increased transfer of glucose to the extracellular space at infection sites. Then, HmSTP1 promotes glucose transport to host cells, facilitating powdery mildew infection (Liu et al., 2024). The carbon battle between plant hosts and pathogens is intense and complex. Pathogens use various strategies to manipulate plant sugar transporters for carbon acquisition. In the future, adopting a sugar starvation strategy to combat pathogens will be a new direction for agricultural resistance.

### 3.3 Sugar transporters are multifaceted mediators in plant-pathogen interactions

In most plants, pathogen invasion significantly induces the expression of sugar transporters in plants (Asai et al., 2016; Chen et al., 2010; Cohn et al., 2014; Cox et al., 2017; Huai et al., 2020; Sun et al., 2021). For instance, *Golovinomyces cichoracearum* infection triggers *AtSWEET12* expression in Arabidopsis leaves, while *Botrytis cinerea* infection enhances *AtSWEET15* expression in Arabidopsis (Chen et al., 2010) and *SISWEET15* in tomato (Asai et al., 2016). However, the function of some sugar transporters in pathogen invasion remains unclear. They may play a minor role in the success of pathogens or provide fuel for plant defense responses. In tomato plants, *Meloidogyne incognita* infection significantly upregulates *STP1*, *STP2*, and *STP12* expression in roots and *STP10* in giant cells, potentially transporting more sugar to phloem parenchyma cells and giant cells to defend against invasion (Sun et al., 2024).

Furthermore, it is surprising that some sugar transporters are significantly downregulated during pathogen infection. Asai et al. (2016) observed a significant downregulation of 21 out of 30 *SISWEETs* in tomato cotyledons infected with gray mold (Asai et al., 2016). Similarly, Breia et al. (2020) found both upregulation and downregulation of various *SWEET* genes in grape berries infected with *Botrytis cinerea*, including *VvSWEET7*, *VvSWEET15*, *VvSWEET2a*, *VvSWEET10*, *VvSWEET11*, *VvSWEET17a*, and *VvSWEET17d* (Breia et al., 2020). The intricate molecular mechanisms underlying these opposing responses remain elusive. One hypothesis is that pathogens may disrupt sugar signaling cascades by downregulating specific *SWEETs*, thereby weakening plant defense mechanisms and creating a favorable environment for their growth and successful infestation (Asai et al., 2016; Breia et al., 2020).

In summary, sugar transporters play diverse roles in plant-pathogen interactions, which highlights their complex functions. Their expression levels fluctuate in response to both bacterial and fungal pathogens. While some sugar transporters facilitate pathogenic invasion, others contribute to plant defense. This further emphasizes the pivotal role of sugar transporters in the intricate interaction between plants and pathogens, highlighting the need for further research to elucidate their precise function and regulation in plant defense. The exact mechanism of sugar transporters, including their preference for sugar substrates, direction of sugar transport, and regulatory factors, still needs to be fully elucidated.

## 4 Sugar transporters as carbon mediators in plant-beneficial microbe symbiosis

Beyond their role in plant-pathogen interactions, sugar transporters, especially SWEET proteins, orchestrate symbiotic relationships between plants and beneficial microorganisms, such as rhizobia, BS and AM fungi, by modulating carbon allocation in the rhizosphere (**Table 1**) (Bezrutczyk et al., 2018; Garcia et al., 2016; Kryukov et al., 2021; Wu et al., 2024a). SWEETs regulate microbial colonization in the rhizosphere by altering sugar distribution in roots and the surrounding soil environment. For example, AtSWEET2, AtSWEET4, AtSWEET11, and AtSWEET12 in Arabidopsis influence microbial dynamics by controlling the spatial availability of sugar (Loo et al., 2024).

### 4.1 Sugar transporters in legume-rhizobia symbiosis

The role of sugar transporters is prominently illustrated in legume-rhizobia symbiosis, a mutualistic interaction where rhizobia colonize root nodules, exchanging fixed nitrogen for the host-derived carbon (Bezrutczyk et al., 2018; Hennion et al., 2019). Rhizobial infection induces the expression of specific *SWEET* genes in legumes (**Figure 2**), highlighting their importance in symbiotic carbon exchange (Hennion et al., 2019; Mergaert et al., 2020).

In *Medicago truncatula*, rhizobial infection significantly upregulates *MtSWEET11* in infected root cells (**Figure 2**) (Kryvoruchko et al., 2016). Functional studies confirm that MtSWEET11 transports sucrose, establishing its role as a key facilitator of carbon supply to rhizobia (Kryvoruchko et al., 2016). Notably, the *Mtsweet11* mutant exhibits no major defects in nitrogen fixation, likely due to functional redundancy within the SWEET family (Kryvoruchko et al., 2016). This redundancy suggests collaborative carbon redistribution among SWEET members to ensure successful symbiosis. Similarly, in *Lotus japonicus*, *LjSWEET3* expression peaks in the vascular tissue of mature nodules (**Table 1**, **Figure 2**) (Sugiyama et al., 2017). Silencing *LjSWEET3* function does not impair nodulation or nitrogen fixation, further emphasizing compensatory mechanisms within SWEET networks (**Table 1**) (Sugiyama et al., 2017).

In soybean (*Glycine max*), it has been demonstrated that the sucrose transporter GmSUT1, localizes to nodule vascular bundles and fixation zones, facilitating sucrose transport from roots to nodules (**Figure 2**) (Deng et al., 2022). Overexpression of *GmSUT1* increases both nodule number and biomass, emphasizing its role in enhancing symbiotic efficiency (Deng et al., 2022).

### 4.2 The regulatory role of SWEET in carbon exchange between plants and *Bacillus subtilis*

In addition to their roles in legume-rhizobia symbiosis, SWEETs also mediate interactions between plants and BS, a well-characterized PGPR (Wu et al., 2024a). BS enhances crop health by forming biofilms and secreting antibiotics, thereby suppressing root diseases (Arnaouteli et al., 2021; Gouda et al., 2018). However, successful root colonization by BS relies on root-secreted sugar, which serves as a carbon source for microbial growth (Arnaouteli et al., 2021).

Recent studies reveal that the AtSWEET2 sugar transporter in Arabidopsis plays a key role in regulating BS colonization (Wu et al., 2024a). The transcription factor AHL29 negatively regulates *AtSWEET2* expression, reducing vacuolar hexoses storage and increasing the hexose efflux into the rhizosphere (**Figure 2**). This enhanced sugar availability promotes BS colonization on roots, highlighting a sophisticated mechanism by which plants modulate microbial interactions through sugar transport (Wu et al., 2024a). The interplay between AtSWEET2 and AHL29 underscores the importance of sugar transporters in shaping plant-microbe interactions.

### 4.3 SWEETs: bridging carbon flow in plant-mycorrhizal fungi symbiosis

During plant-mycorrhizal fungi symbiosis, *Glomeromycota* fungi form mutually beneficial AM associations with plant roots, facilitating nutrient exchange (Garcia et al., 2016; Wipf et al., 2019). In *Medicago truncatula*, for example, this symbiosis is characterized by efficient carbohydrate provision to AM fungi and effective phosphate extraction in return (Garcia et al., 2016; Wipf et al., 2019). Although SUTs have been found to affect mycorrhization (Bitterlich et al., 2014), research on sugar transporters that affect AM symbiosis mainly focuses on SWEET proteins (An et al., 2019; Manck-Götzenberger and Requena, 2016; Tamayo et al., 2022; Zhao et al., 2019; Zheng et al., 2024).

Significant changes in *SWEET* family gene expression have been observed during AM fungi colonization. Specifically, MtSWEET1b, located on the peri-arbuscular membrane of cortical cells, experiences significant induction in cells containing clumps (**Figure 2**) (An et al., 2019). Disruption of *MtSWEET1b* function leads to the collapse of AM fungi (An et al., 2019). In soybeans, transcriptome data indicate a considerable elevation in *GmSWEET6* and *GmSWEET15* expression in cells colonized by AM fungi (Zhao et al., 2019). Further studies have shown that GmSWEET6 is involved in AM symbiosis and mediates sucrose efflux to fungi (**Figure 2**) (Zheng et al., 2024). In potato (*Solanum tuberosum*), the SWEET family comprises 35 *StSWEET* genes, and 22 are differentially regulated in response to AM symbiosis (Manck-Götzenberger and Requena, 2016). StSWEET7a, located on the plasma membrane, specifically relocates to arbuscular-containing root cells. Overexpression of *StSWEET7a* in potato roots increases glucose, fructose, and mannose content in cells, and plants are more rapidly colonized by AM fungi (**Figure 2**) (Tamayo et al., 2022).

In brief, SWEETs that transport sucrose in leguminous plants are induced by rhizobia and highly expressed in nodules. Additionally, SWEETs maintain a mutualistic relationship between plants and BS by regulating sugar secretion. SWEETs are also involved in the specific transport of sugars from host plants to symbiotic AM fungi, facilitating glucose and fructose transport across the peri-arbuscular membrane, positively influencing mycelial growth and fungal biomass. Although the primary SWEET transporter remains undetermined in these cases, the redistribution of carbon sources, represented by sugars, is crucial for establishing symbiotic relationships between plants and beneficial microorganisms. However, it is still unclear whether plants and beneficial microorganisms share a common sugar transport pathway.

## 5 Molecular strategies for sugar transporter regulation during plant-microbe interactions

Recent research has shed light on the intricate molecular mechanisms governing carbon flux through various sugar transporters during plant-microbial interaction (Xu et al., 2024; Yamada et al., 2016; Wu et al., 2024a, b). These findings are attributed to the dual roles played by these sugar transporters in pathogen susceptibility and resistance as well as beneficial symbioses. Notably, remarkable progress has been made in elucidating the transcriptional and post-transcriptional regulation of these sugar transporters.

### 5.1 Pathogenic TALEs: manipulators of sugar transporters expression

Transcription activator-like effectors (TALEs) are a distinct group of proteins secreted by bacterial pathogens through their Type III secretion system during host-pathogen encounters (Zhang et al., 2022). These proteins bind to promoters, thereby profoundly influencing the expression of several genes encoding SWEET sugar transporters (**Figure 2**) (Blanvillain-Baufumé et al., 2017; Elliott et al., 2024; Xu et al., 2019, 2024; Zárate-Chaves et al., 2023). Specifically, in rice, TALEs bind to promoters, triggering the expression of *SWEETs* sugar transporters such as *OsSWEET11* (*Xa13/Os8N3*), *OsSWEET14* (*Os11N3*), and *OsSWEET13* (*Xa25*) during interactions with *Xoo* (Blanvillain-Baufumé et al., 2017; Xu et al., 2019, 2024). In a recent study, silencing of TALEs has been found to inhibit *MeSWEET10a* expression in cassava (Elliott et al., 2024; Zárate-Chaves et al., 2023). This highlights the significant role played by TALEs in modulating the expression of SWEETs sugar transporters, which are emerging as key players in host-pathogen dynamics.

### 5.2 Transcriptional orchestration of sugar transporters in defense and symbiosis

Currently, there are few reports on the transcriptional regulation mechanisms in plants in response to pathogens or beneficial microorganisms by targeting genes that encode sugar transporters. However, existing evidence suggests that plants can regulate the expression of sugar transport-related genes through transcription factors induced by signaling molecules such as sugars and plant hormones, thereby regulating sugar transport and reallocating sugar distribution (Chen et al., 2016; Kamranfar et al., 2018; Kim et al., 2021; Li et al., 2018; Ma et al., 2017; Zhang et al., 2023). Multiple transcription factors have been identified as regulatory factors for sugar transport-related genes, mediating various biological processes related to sugar transport. These factors include NAC (NAM, ATAF1/2, CUC2) in Arabidopsis and rice (Kamranfar et al., 2018; Ren et al., 2021), NF-YC12, NF-YB1, and GRF (Growth Regulating Factors) in rice (Li et al., 2018; Xiong et al., 2019), the Dof (DNA Binding One Finger) family in rice (Kim et al., 2021; Wu et al., 2018), the BZIP (Basic Leucine Zipper) family in Arabidopsis and soybeans (Chen et al., 2016; Song et al., 2013), the MYB family in apple (*Malus domestica*), chicory (*Cichorium intybus*) and rice (Wei et al., 2017; Wang et al., 2021b; Zhang et al., 2023), and the ABA-responsive transcription factor MdAREB2 in apple and tomato (Ma et al., 2017; Zhu et al., 2023).

Recent studies have identified that AHL29, a transcriptional repressor belonging to the AT-Hook Motif Containing Nuclear Localized (AHL) transcription factor family in Arabidopsis, promotes BS colonization in plant roots by repressing the expression of *AtSWEET2* (**Figure 2**) (Wu et al., 2024a). Despite these findings, our understanding of how plants precisely regulate their transcriptional response to pathogens or beneficial microorganisms by targeting genes encoding sugar transporters remains incomplete.

### 5.3 Post-translational modifications: fine-tuning of sugar transporter function

The post-translational control of sugar transporters includes oligomerization, protein-protein interactions, phosphorylation, ubiquitination, etc., all of which affect the affinity and transport capacity of sugar transporters (Anjali et al., 2020). Many studies have shown that homologous oligomerization is crucial for the sugar transport activity of sugar transporters and might constitute a conserved regulatory mechanism in various plants (Anjali et al., 2020; Krügel et al., 2008; Garg et al., 2020; Xuan et al., 2013). Heteromeric oligomerization weakens their sugar transport activity, as exemplified by heterodimers or polymers formed between VvSUC11, VvSUC12 and VvSUC27 in grapes (Cai et al., 2021).

Meanwhile, sugar transporters in plants engage in dynamic interactions with other proteins, fine-tuning their transport functions (Anjali et al., 2020). For instance, AtSUT4 interacts with five cytochrome b5 family proteins in Arabidopsis (Li et al., 2012), StSUT4 interacts with the ethylene receptor ETR2 and calmodulin-1 (PCM1) in potato (Garg et al., 2022), StSWEET11 interacts with StSP6A in potato (Abelenda et al., 2019), and GmSWEET10a interacts with Dt1 in soybean (Li et al., 2024). These protein-protein interactions enhance the dynamic and adaptable nature of sucrose transport in plants, responding to various environmental and developmental signals.

Moreover, phosphorylation plays a pivotal role in modulating the activity of plant sugar transporters (Anjali et al., 2020). In Arabidopsis, the phosphorylation of the 485th threonine residue (T485) of AtSTP13 by Brassinosteroid insensitive-associated kinase 1 (BAK1) enables plants to adopt a “food restriction” strategy as a defense mechanism against invading pathogens, thereby enhancing their disease resistance (Yamada et al., 2016). WALL-ASOCIATED KINASE LIKE 8 (WAKL8) phosphorylates AtSUC2, enhancing sugar transport activity (Xu et al., 2020). SnRK2 kinase catalyzes the phosphorylation of AtSWEET11 and AtSWEET12, enhancing their sucrose transport activity and promoting root growth under drought stress (Chen et al., 2022). In tomato, calcium-dependent protein kinase (CPK) enhances drought resistance by phosphorylating the sugar transporter TST2 (Zhu et al., 2024).

In addition to phosphorylation, ubiquitination also plays a role in regulating the activity of sugar transporters (Wu et al., 2024b). The potato StRFP1 protein ubiquitinates and degrades StSWEET10c and StSWEET11 in a 26S proteasome-dependent manner, enhancing the potato resistance to *Phytophthora infestans* (**Figure 2**) (Wu et al., 2024b). Although there are few studies on the post-translational regulation of glucose transporters in the plant-microorganism interaction, plants can indeed regulate the activity of glucose transporters at the protein level. Regulating the oligomerization, phosphorylation, and ubiquitination of sugar transporters, as well as manipulating the expression of interacting proteins, may provide a new pathway to alter sugar allocation.

## 6 Conclusions and perspectives

In recent years, the pivotal roles of leaf and rhizosphere microbiota in plant growth, stress tolerance, and nutrient utilization have garnered considerable attention (Glick and Gamalero, 2021; Udvardi and Poodle, 2013; Wipf et al., 2019). Carbohydrates, primarily sugars derived from plant photosynthesis, serve as crucial energy and nutrient sources for microorganisms, with their dynamic transportation and redistribution playing a central role in shaping plant-microbe interactions (Bezrutczyk et al., 2018; Hartmann et al., 2020). These processes not only influence the infectivity of pathogens but also determine the efficiency of beneficial microbial symbioses (Hartmann et al., 2020; Oliva and Quibod, 2017).

Sugar transporters, including MSTs, SUTs, and SWEETs, are key mediators of carbohydrate allocation, ensuring precise sugar delivery to sink tissues and organs (Braun, 2022; Chen et al., 2015a, 2024). These transporters are indispensable for maintaining carbohydrate homeostasis during plant-microbe interactions, enabling effective source-sink communication and modulating both pathogenic and symbiotic outcomes (Chen et al., 2024; Devanna et al., 2021; Kryukov et al., 2021).

Optimizing the expression and activity of sugar transporters through genetic engineering or agronomic practices holds immense potential for enhancing plant stress tolerance, survival rates, and adaptability (Blanvillain-Baufumé et al., 2017; Elliott et al., 2024; Gao et al., 2018). Molecular breeding approaches can identify genotypes with enhanced sugar transport capabilities, paving the way for crop varieties with improved stress resistance, higher yields and efficient symbiotic relationships. Priority should be given to exploring allelic variations that confer disease resistance or facilitate beneficial microbial interactions.

Functional redundancy among sugar transporters poses a significant challenge in deciphering their roles. For instance, despite the high expression of *LjSWEET3* and *MtSWEET11* in root nodules, their knockout mutants exhibit no significant phenotypic changes, likely due to compensatory mechanisms (Sugiyama et al., 2017; Kryvoruchko et al., 2016). To overcome this, future studies should employ strategies such as generating multiple gene knockouts, integrating transcriptomic and metabolomic analyses, and conducting genetic complementation experiments to unravel the complex regulatory networks governing sugar transporters.

In the future, several promising avenues warrant further exploration: (1) Identifying the specific sugars transported during plant-microbe interactions; (2) Determining the precise subcellular localization of sugar transporters to enable targeted manipulation; (3) Elucidating the molecular pathways that regulate sugar transporter activity and expression; (4) Investigating how functional redundancy is achieved and its implications for plant-microbe dynamics.

Sugar transporters are pivotal in shaping plant-microbe interactions and hold transformative potential for sustainable agriculture. By leveraging advanced molecular tools and breeding strategies, we can unlock the full potential of these transporters to develop resilient, high-yielding crop varieties. This will not only enhance agricultural productivity but also contribute to eco-friendly farming practices, ensuring food security in the face of global challenges.
